# Sex Differences in the Atherogenic Risk Index in Healthy Mexican Population and Its Relationship with Anthropometric and Psychological Factors

**DOI:** 10.3390/jpm13101452

**Published:** 2023-09-29

**Authors:** Aniel Jessica Leticia Brambila-Tapia, Ingrid Patricia Dávalos-Rodríguez, Carlos Adán Méndez-García, Frida Isadora Bárcenas-Robles, Itzae Adonai Gutiérrez-Hurtado

**Affiliations:** 1 Departamento de Psicología Básica, Centro Universitario de Ciencias de la Salud (CUCS), Universidad de Guadalajara, Guadalajara 44340, Jalisco, Mexico; 2 División de Genética, Centro de Investigación Biomédica de Occidente (CIBO), Instituto Mexicano del Seguro Social (IMSS), Guadalajara 44340, Jalisco, Mexico; ingriddavalos@hotmail.com; 3 Departamento de Biología Molecular y Genómica, Centro Universitario de Ciencias de la Salud (CUCS), Universidad de Guadalajara, Guadalajara 44340, Jalisco, Mexico; 4 Licenciatura en Médico, Cirujano y Partero, Centro Universitario de Ciencias de la Salud (CUCS), Universidad de Guadalajara, Guadalajara 44340, Jalisco, Mexico; carlos.mendez4429@alumnos.udg.mx (C.A.M.-G.); frida.barcenas7678@alumnos.udg.mx (F.I.B.-R.)

**Keywords:** dyslipidemia, atherogenic risk index, sex, psychological factors, quality of food intake

## Abstract

Dyslipidemia is a risk factor for cardiovascular disease and mortality; however, the association of this variable with a wide range of personal and psychological variables has not been researched. Therefore, the aim of this study was to compare lipid levels and anthropometric measures between sexes and to determine the association between personal and psychological variables with the atherogenic risk index (ARI). An adult population which auto-reported as healthy was invited to participate via social media and in person. They filled out a questionnaire with personal and psychological variables; in addition, the body mass index (BMI) and waist-to-hip ratio (WHR) were measured, and a blood sample was obtained to determine serum lipids. A total of 172 participants were included, from which 92 (53.49%) were women; both sexes were comparable in age and most sociodemographic values. Men showed significantly higher levels of total cholesterol, LDL cholesterol, triglycerides, ARI, and lower levels of HDL cholesterol. The men also showed higher values of WHR than the women. In the bivariate analysis, ARI showed the highest correlation with WHR (r = 0.664) in the men and with BMI (r = 0.619) in the women. In the multivariate analysis, the quality of food intake was negatively correlated with ARI in the global and women’s samples, and the psychological variables of assertiveness and positive relations with others were negatively correlated with ARI in women, while purpose in life was negatively correlated with ARI in men. In conclusion, the higher levels of serum lipids and ARI in men can be explained by the higher values of WHR in this sex. Behavioral and psychological variables could be protective factors for high ARI.

## 1. Introduction

Dyslipidemia consists of increased total cholesterol, low-density lipoprotein (LDL) cholesterol and triglyceride levels, and decreased levels of high-density lipoprotein (HDL) cholesterol [1]. To date, there is increasing evidence of a relationship between physical and psychological stress and cardiovascular disease [2,3]. Among the factors measured as psychological stress are stressful life events [1], job stress [4], and general perceived stress [5]. The relationship between psychological stress and dyslipidemia has been proposed to be due to increased activity in the hypothalamic–pituitary–adrenal (HPA) axis, resulting in increased cortisol levels, insulin resistance, and abdominal obesity; with the consequent increase in lipids and blood pressure, all these changes increase the likelihood of presenting metabolic syndrome [6].

An association has been shown between hypertriglyceridemia and stressful life events in men [1], and between job stress and dyslipidemia [2]. In addition, a meta-analysis, including 17 studies, showed an association between stress and body mass index (BMI), waist circumference, serum triglyceride levels, HDL, and diastolic blood pressure [7].

In addition, sex differences in cardiovascular risk factors have been observed, with men showing higher central adiposity [8,9] and higher cholesterol levels than women [10], although some evidence has shown that these differences tend to diminish after the age of 50 years old [9]. Nevertheless, none of these studies were performed in a relatively healthy population, and nor did they study the relationship between anthropometric variables and lipid profile in the same study, with analyses separated by sex. Therefore, studies addressed to identify these possible relationships are needed.

In addition, there are no studies in which many positive and negative psychological variables, different from stress, are measured in their association with serum lipids. These analyses would shed light on the relationship between psychological variables and serum lipid levels in healthy populations and could generate new studies that prevent the appearance of dyslipidemia and cardiovascular problems.

Therefore, the objectives of this study were to compare the anthropometric measures and serum lipids between sexes and to determine the association of psychological variables with the atherogenic risk index (ARI) in a multivariate analysis, adjusted for confounders, in the global sample and separated by sex. Our hypotheses were as follows: (a) there are sex differences in body composition measures, including waist/hip ratio (WHR) and serum lipids, with men showing higher levels of serum LDL cholesterol, triglycerides, and WHR and lower levels of HDL cholesterol; (b) positive psychological variables (i.e., self-acceptance and emotional intelligence) are related with lower levels of total ARI after adjusting for confounders; (c) negative psychological variables (anxiety and depression) are associated with higher levels of ARI after adjusting for confounders.

## 2. Subjects and Methods

### 2.1. Ethical Considerations

The study was conducted according to the guidelines of the Declaration of Helsinki and was approved by the ethical committee of the Health Sciences University Center, with the registration number: 19–21. All the participants signed an informed consent form.

### 2.2. Subjects

The inclusion criteria of the study were as follows: (a) subjects older than 18 years old, (b) subjects without chronic or acute diseases that were known of by the subject (auto-reported), (c) subjects who were not consuming illegal drugs (including marijuana), (d) subjects who were not consuming hormonal products to increase muscular mass, (e) subjects who were not pregnant, (f) subjects who were not genetically related with another participant of the study (i.e., siblings, cousins), and (g) subjects who preferably did not smoke. The elimination criterium was (a) the absence of the measurement of any variable.

#### 2.2.1. Study Design

This is an observational cross-sectional study, by reason of no interventions or follow-up measurements being performed.

#### 2.2.2. Procedures

The study was performed from July to November of 2022. The invitation was conducted through an announcement distributed via social networks (WhatsApp, Facebook) and personally to university students. All the subjects who contacted the research team verified that they met the inclusion criteria (via auto-report). If they accepted to participate, they were processed (in groups from 11 to 20 participants) in the facilities of the university of Guadalajara, where they signed an informed consent and filled in an electronic questionnaire which included personal and psychological variables, all these variables were self-reported.

After filling in the questionnaire, height and weight of participants were obtained by trained students with Tanita brand scales (model bc-533) and a measuring tape attached to the wall, to calculate the BMI. The hip and waist circumferences were also obtained by trained students, by means of a measuring tape; these measurements were used to calculate the waist/hip ratio (WHR). The blood samples (to perform the laboratory tests) were obtained by qualified personnel (three biochemicals) who worked for a certified laboratory. After obtaining the samples, these were transported to a certified biochemical laboratory, where the biochemical analyses were performed by trained personnel.

#### 2.2.3. Sample Size

The sample size was calculated with the correlation’s formula [11], which yielded a total of 47 subjects, as a minimum, to detect a moderate correlation of 0.4 or higher as being significant. However, the minimum sample size intended was 80 individuals per sex.

### 2.3. Personal Variables

The personal and sociodemographic variables included were: sex, age, schooling, whether they had a job, whether they had a romantic partner, having children, socioeconomic level, daily free hours, daily hours of physical activity, monthly extra money divided into five categories (from zero to more than USD 150) and alcohol and smoking consumption frequency (five categories, from never to four or more times in the week). Sleep satisfaction was measured with the first item of the OVIEDO sleep questionnaire, from 1 (very unsatisfied) to 7 (very satisfied); sleep quality was measured with the second item (which in turn consists of five items) of the OVIEDO sleep questionnaire, from 1 to 5 (low quality to high quality) [12]. The quality of food intake was measured with the Mini-Ecca scale, from 1 to 12 (very low quality to very high quality) [13]; finally, two additional questions of eating behavior were included: (a) the frequency of food consumption outside home, and (b) the frequency of food consumption in excess, both questions were measured with seven answer options (from 1: less than once in a month to 7: all the days of the month). These questions were obtained from the eating behavioral questionnaire [14].

### 2.4. Psychological Variables

The following psychological variables were measured: depression, with the 10-items CES-D scale, from 1–4 (no days to every day) [15,16]; anxiety with the Generalized Anxiety Disorder test (GAD-7), from 0 to 3 (never to almost all of the days) [17]; positive and negative emotions with the positivity-self scale (PSS), from 1 to 5 (never to almost always) [18]; the six subscales of the shortened version of the psychological well-being (PWB) scale (self-acceptance, autonomy, environmental mastery, personal growth, positive relations with others, and purpose in life), measured from 1 to 6 (totally disagree to totally agree) [19]; optimism was measured with the Life Orientation Test (LOT-R), from 1 to 5 (totally disagree to totally agree) [20]. Additionally, we measured five–six items of four subscales of the Trait Emotional Intelligence Questionnaire (TEIQUE): self-motivation (five items), emotion perception (five items), assertiveness (six items), and emotion regulation (six items), from 1 to 7 (totally disagree to totally agree) (these items are described in Supplementary file S1) [21].

### 2.5. Serum Lipids Measurement

The serum lipids measured were complete lipid profile test, including total cholesterol, low-density lipoprotein (LDL), high-density lipoprotein (HDL), and triglycerides. These analyses, which were performed in a certified biochemical laboratory, involved the sample being drawn into red-top tubes. Then, the serum was obtained via centrifugation and refrigerated immediately and finally, the analyses were performed on the same day with standardized reagents and equipment using the colorimetry method. With these variables, we obtained the ARI via the division of total cholesterol by HDL cholesterol.

### 2.6. Statistical Analysis

In order to describe the numerical variables, we used mean and standard deviations when the distribution was parametric, and median and ranges when it was non-parametric. To compare sociodemographic variables between sexes, we used chi-squared test for qualitative variables and Student’s *t*-test or Mann–Whitney U test for quantitative ones (depending on whether the distribution was parametric or non-parametric). In order to correlate quantitative variables with serum lipids, we used Pearson’s and Spearman;s correlation tests, depending on the parametric or non-parametric distribution of the data. In addition, multiple linear regression analysis (using the stepwise method) with the ARI as dependent variable was performed for the whole sample and segmented by sex.

Finally, the Cronbach´s alpha test was obtained for all the psychological instruments (including the subscales) in order to obtain the reliability of each scale and subscale applied. All analyses were performed with the software SPSS v.25, and a *p* value < 0.05 was considered as significant.

## 3. Results

A total of 172 participants were included, from which 92 (53.49%) were women, the mean ± SD of age of the whole sample was: 27.14 ± 10.66. All instruments employed had a Cronbach´s alpha ≥ 0.6.

The descriptive data of sociodemographic and psychological variables are shown in Table 1, where we observe that all sociodemographic variables were similar between sexes with the exception of daily free hours, which were higher in men than in women (Table 1). For psychological variables, we observed that anxiety, depression, and negative emotions were significantly higher in women than in men, and that autonomy, assertiveness, and positive emotions were significantly higher in men than in women (Table 1).

In the comparison of anthropometric measures and serum lipids between sexes, we observed that BMI was similar between sexes; however, WHR was very significantly higher (*p* = 7.35 × 10^−13^) in men than in women. In addition, total cholesterol, LDL cholesterol, triglycerides, and ARI were also significantly higher in men than in women (Table 2). When we categorized the variables, serum lipids and anthropometric measures in normal or high levels, according to the normal laboratory values (for serum lipids) and to the accepted international values for BMI [22] and desirable WHR for the Mexican population [23], we observed that men showed significantly higher levels of LDL cholesterol, triglycerides, ARI, and WHR than women (Table 3).

In the bivariate analysis between personal and sociodemographic variables with ARI, we observed positive significant correlations with BMI and WHR in both sexes, being higher between WHR and ARI in men (r = 0.664, *p* <0.01) and between BMI and ARI in women (r = 0.614, *p* < 0.01). The sociodemographic variables age, schooling, and having children also showed low to moderate positive significant correlations with ARI in both sexes (Table 4). The behavioral variable “frequency of food consumption in excess” showed a low positive correlation with ARI only in women (r = 0.332, *p* < 0.01), while the psychological variable “autonomy” showed a low positive correlation with ARI only in men (r = 0.242, *p* < 0.05) (Table 4). We also observed a positive moderate correlation between age and WHR in men and women: r = 0.617, *p* < 0.01 and r = 0.449, *p* < 0.01, respectively.

In the multivariate regression analyses, we observed for the whole sample that the variable WHR was the most associated (positively) with ARI, followed by BMI, while the psychological variable “autonomy” was positively correlated with ARI, and “quality of food intake” was negatively correlated (with a borderline p value) with ARI (Table 5).

In the multivariate regression analysis in women, we observed that BMI was the variable most associated (positively) with ARI, followed by age and daily free hours; schooling was also positively correlated with ARI. The psychological “variables positive relations with others” and “assertiveness” were negatively correlated with ARI, while “positive emotions” was positively correlated with ARI. Finally, quality of food intake was marginally correlated (negatively) with ARI (Table 6).

In the multivariate regression analysis for men, we observed that WHR was the variable most correlated (positively) with ARI. We also observed that the psychological variables “autonomy” and “emotional regulation” were positively associated with ARI, while “purpose in life” was negatively correlated with ARI. Finally, monthly extra money was marginally correlated (negatively) with ARI (Table 7).

## 4. Discussion

Dyslipidemia has been associated with mortality by cardiovascular disease in large studies [24,25]; this being more frequently documented in men than in women [10]. Men have also shown higher waist circumference and WHR in different studies when compared to women [8,9,26], which represents higher abdominal adiposity [8]. These differences have been explained by the protective effect that estrogens exert for dyslipidemia in women during the reproductive years and 10 years after the onset of menopause in comparison with men [27]. Although many reports have documented differences in adiposity and dyslipidemia between sexes [8,9,10,26], we did not find a study that studied these measurements in a sample of relatively healthy population, and also intended to relate the ARI with a wide range of personal and psychological variables in each sex.

In this study, we could corroborate the first hypothesis, showing that men had significantly higher levels of total cholesterol, LDL cholesterol, triglycerides, and ARI than women, as well as lower levels of HDL cholesterol. Additionally, we found very significantly higher levels of WHR in men when compared with women, despite no differences in BMI being found (Table 2). These results coincide with previous reports showing these findings [8,9,10,26], and suggest that the abdominal adiposity is the main contributing factor for dyslipidemia in men, which coincides with the fact that the liver, located in the abdomen, is the organ responsible for the metabolism of cholesterol [28]. These findings also coincide with previous reports mentioning that WHR is the best anthropometric predictor of cardiovascular disease in both sexes [29,30]. Our results, together with the reported literature, emphasize the importance in WHR reduction (mainly in men) in order to decrease the risk of developing dyslipidemia, cardiovascular disease, and subsequent death. The higher levels of serum lipids in men are also corroborated when these variables were categorized into high or normal (according to the laboratory ranges). We observed a higher frequency of high levels of total cholesterol, LDL cholesterol, triglycerides, and ARI in men. HDL cholesterol did not show differences by considering that normal values according to the laboratory parameters are much higher in women than in men. The WHR categorization, according to the desirable values for Mexican population, also showed a higher frequency of high WHR in men than in women.

With respect to the correlations between sociodemographic and psychological variables with ARI in each sex, we observed that among the sociodemographic variables: age, schooling, and having children were positively correlated with ARI in both sexes. In the case of age, this correlation is in part explained by the positive correlation between age and WHR in both sexes. Additionally, age could be also related with a lower frequency of a healthy lifestyle, that could also increase ARI. In relation to the positive correlations between ARI with schooling and having children, we suggest that this can be explained by the positive moderate correlation between these variables and age in both sexes (with a correlation in the whole sample of r = 0.549, *p <* 0.001 and r = 0.659, *p* < 0.001, respectively). In relation with the anthropometric variables, men showed a higher correlation between ARI and WHR (r = 0.664, *p* < 0.01) and for women, between ARI and BMI (r = 0.614, *p* < 0.01). This can be explained by the fact that men showed significantly higher levels of WHR than women; however, these findings contrast with the literature, which explains that WHR is the best anthropometric predictor for cardiovascular disease in both sexes [29,30]. These discrepancies are explained by considering that we did not measure the risk of developing cardiovascular disease, which could require a longitudinal study with a much larger sample size and a long follow up.

Following with the bivariate correlations, we observed that the frequency of food consumption in excess was positively correlated with ARI only in women (r = 0.332, *p* < 0.05); this finding coincides with a previous report performed in Mexican university students, which found an increased odds ratio (OR) of having high LDL cholesterol with this variable [31]. However, this report did not perform correlations separated by sex nor used the ARI that englobes LDL cholesterol and triglycerides. Finally, in the bivariate analysis, the variable “autonomy” was the only psychological variable correlated with ARI, but only in men. We explain this relationship by considering that “autonomy” is a variable related to the ability of being less influenced by others’ opinions in the person´s life. Therefore, it is possible that men who had higher levels of “autonomy” (Table 1), a difference that could be explained via cultural and biological factors, are less prone to follow dietary and lifestyle recommendations.

In the multivariate analysis for the whole sample, we obtained a robust model (r = 0.661) where WHR was the most associated variable with ARI, followed by BMI, autonomy, and quality of food intake, which was negatively correlated with ARI (Table 5). This analysis highlights the influence of WHR (more than BMI) in ARI, as well as the positive correlation of the psychological variable “autonomy” on ARI in both sexes. In addition, quality of food intake appears as a protective variable against higher values of ARI in both sexes.

In the multivariate analysis for women, besides the variables significantly correlated in the bivariate analysis with ARI (including BMI, age and schooling), we observed that daily free hours were positively correlated with ARI. This last correlation can be explained by considering that daily free hours can be related to more sedentarism and food consumption. In addition, the psychological variables “positive relations with others” and “assertiveness” were negatively correlated with ARI. These correlations can be explained by the positive effect that social support could exert on healthy behaviors and the negative correlation with assertiveness, and can be explained by the influence of this variable, related to the ability to defend one’s own opinions and rights, on healthy lifestyles, probably by avoiding the social influence of unhealthy eating habits on a person´s life. However, for these two psychological variables, a bilateral relationship can also be found, via considering the beneficial influence that these variables could exert in the hypothalamic–pituitary–adrenal axis. This is because it has been shown that chronic elevated cortisol levels increase fat deposits, lipolysis, circulating fatty acids, and appetite [6,32,33]. In this sense, the variables of “positive relations with others” and “assertiveness” could be avoiding chronic stress and cortisol secretion. Finally, in the multivariate regression analysis for women, we also found that positive emotions were positively correlated with ARI. This observation is an unexpected finding and can be explained by a possible protective effect of serum lipids in neuroinflammation and serotoninergic neurotransmission, which has been recently proposed in light of the findings that low serum cholesterol levels are associated with suicidal behavior in patients with depressive disorders [34]. This explanation could be plausible in the population of women in this study, because only three (3.33%) women showed high ARI, indicating that most of the women were within normal ARI ranges. However, more evidence is needed in order to support these hypotheses.

In the multivariate regression analysis in men, we observed that besides the variables associated with ARI in the bivariate analysis (WHR and autonomy), the psychological variable “purpose in life” (measured with questions like “I have a sense of direction and purpose in life”) was negatively correlated with ARI, while “emotion regulation” (questions like “I know how to snap out my negative moods”) was positively correlated with it. Although these findings need to be further explored with larger sample sizes, it is possible that men with higher values of purpose in life are more occupied in their goals, which diminishes unhealthy lifestyles related to higher ARI. In the case of the positive correlation between “emotion regulation” with ARI, this could suggest that the reason that men consider themselves to have a high emotional management of negative emotions is because they experience those emotions more frequently; additionally, it could be related to the repression of negative moods. Both of these possibilities could increase cortisol levels and fat deposits resulting in increased abdominal obesity. Finally, the variable monthly extra money was marginally correlated (negatively) with ARI; this finding can be explained by the positive influence of a higher income on a healthier nutrition.

Interestingly, we could not verify the third hypothesis, because depression and anxiety were not positively related with ARI in the global sample or segmented by sex. These results do not coincide with the positive associations previously reported between dyslipidemia and stress [3,4,5], a variable related with anxiety and depression; however, these discrepancies can be due to the small sample size in this study and/or because it is possible that only perceived stress, job stress, and stressful life events are associated with dyslipidemia, and not anxiety or depression.

The main limitation of this study is the relatively small sample size when compared with other larger reports; likewise, the cross-sectional nature of the design does not permit the inference of causal relationships. In addition, the measurement of perceived stress would have permitted us to identify the role that this variable plays in ARI after controlling for the rest of the variables. However, we consider that the inclusion of many personal and psychological variables, along with all serum lipids and ARI, together with the measurement of BMI and WHR, permitted us to obtain interesting results in relation to each sex that could shed light on dyslipidemia research and give rise to new useful studies that could corroborate these results.

In conclusion, we detected higher levels of total cholesterol, LDL cholesterol, triglycerides, and ARI, and lower levels of HDL cholesterol in men when compared with women, a difference that can be attributed to the higher values of WHR in men. This confirms that abdominal adiposity is the main risk factor for dyslipidemia and is higher in men than in women. In addition, other psychological, personal, and behavioral factors were associated with ARI in the global sample and by each sex, emphasizing the importance of quality of food intake as a protective factor in both sexes, and of the psychological variables assertiveness and positive relations with others in women, and purpose in life in men, which were negatively correlated with ARI in the multivariate analyses by sex. Furthermore, larger and prospective studies are needed in order to confirm these results.

## Figures and Tables

**Table 1:** Descriptive data of sociodemographic and psychological variables.

Variable	Women (n = 92)	Men (n = 80)	*p* Value
Age	27.85 ± 10.45	26.33 ± 10.91	0.101
With romantic partner, n (%)	46 (50.0)	43 (53.75)	0.649
With children, n (%)	24 (26.1)	11 (13.75)	0.057
With job, n (%)	51 (55.43)	38 (47.50)	0.359
Schooling, n (%) - Elementary school - Secondary - Preparatory - University (Bachelor’s degree) - Master’s degree - Ph.D. degree	1 (1.1) 3 (3.3) 49 (53.2) 32 (34.8) 7 (7.6) 0 (0.0)	0 (0.0) 2 (2.5) 50 (62.5) 21 (26.3) 6 (7.5) 1 (1.2)	0.636
Socioeconomic level, n (%) - Very low - Low - Average - High - Very high	0 (0.0) 17 (18.5) 74 (80.4) 1 (1.1) 0 (0.0)	2 (2.5) 15 (18.7) 59 (73.8) 4 (5.0) 0 (0.0)	0.194
Monthly extra money, mean ± SD	2.93 ± 1.18	3.20 ± 1.37	0.145
Smoking frequency, median (range)	0 (0–4)	0 (0–4)	0.294
Alcohol consumption frequency, mean ± SD	1.47 ± 0.87	1.44 ± 0.94	0.906
Daily free hours, median (range)	4 (0–11)	4 (0–14)	0.010*
Daily physical activity hours, median (range)	1 (0–5)	1 (0–4)	0.380
Sleep satisfaction (OVIEDO scale), median (range)	3.89 ± 1.44	4.19 ± 1.54	0.360
Sleep quality (OVIEDO scale), mean ± SD	3.56 ± 0.96	3.74 ± 0.93	0.206
Frequency of food consumption outside home	3.93 ± 1.38	4.69 ± 1.35	0.001 *
Frequency of food consumption in excess	3.50 ± 1.60	3.64 ± 1.33	0.561
Quality of food intake (Mini-Ecca scale), mean ± SD	7.71 ± 2.64	7.19 ± 2.39	0.180
**Psychological variables**			
Anxiety (GAD-7), mean ± SD	1.19 ± 0.74	0.90 ± 0.60	0.018 *
Depression (CES-D), mean ± SD	1.95 ± 0.58	1.78 ± 0.45	0.031 *
Psychological wellbeing (PWB), mean ± SD - Self-acceptance - Autonomy - Purpose in life - Positive relations with others - Personal growth - Environmental mastery	4.60 ± 1.23 3.89 ± 0.99 4.54 ± 1.22 4.89 ± 1.04 5.10 ± 0.97 4.38 ± 1.10	4.83 ± 1.04 4.35 ± 0.98 4.65 ± 1.13 4.72 ± 0.99 5.01 ± 0.92 4.47 ± 0.98	0.256 0.003 * 0.613 0.213 0.317 0.539
Emotional intelligence (TIEQUE), mean ± SD - Assertiveness - Emotion regulation - Self-motivation - Emotion perception	4.65 ± 0.97 4.84 ± 1.16 5.17 ± 1.19 4.92 ± 1.45	5.13 ± 1.10 5.04 ± 1.26 4.97 ± 1.20 5.06 ± 1.38	0.003 * 0.297 0.280 0.530
Positive emotions (PSS), mean ± SD	3.65 ± 0.65	3.83 ± 0.52	0.049 *
Negative emotions (PSS), mean ± SD	2.62 ± 0.65	2.39 ± 0.57	0.016 *
Optimism (LOT-R), mean ± SD	3.67 ± 0.74	3.70 ± 0.67	0.848

* *p* Value obtained with Chi-squared test, Student’s *t*-test and Mann–Whitney U test. Monthly extra money: five categories, from nothing to more than USD 150; smoking and alcohol consumption frequency were measured from 0 to 4 (never to more than 4 times in the week); sleep satisfaction (OVIEDO scale), from 1 to 7 (very unsatisfied to very satisfied); sleep quality (OVIEDO scale), from 1 to 5 (low quality to high quality); quality of food intake (Mini-Ecca scale) from 1 to 12 (very low quality to very high quality); frequency of food consumption outside home and frequency of food consumption in excess, from 1 to 7 (less than once in a month to all the days); anxiety (GAD-7 scale), from 0 to 3 (never to almost all the days); depression (CES-D scale), from 1 to 4 (none day to all the days); subscales from psychological wellbeing (PWB), from 1 to 6 (totally disagree to totally agree); emotional intelligence (TEIQUE scale), from 1 to 7 (totally disagree to totally agree); positive and negative emotions (PSS scale), from 1 to 5 (never to almost always); optimism (LOT-R), from 1 to 5 (totally disagree to totally agree).

**Table 2:** Comparison of anthropometric variables and serum lipids between sexes.

Variable, Mean ± SD	Global (n = 172)	Women (n = 92)	Men (n = 80)	*p* Value
Total cholesterol	170.96 ± 30.06	165.95 ± 29.10	176.71 ± 36.43	0.033 *
LDL cholesterol	101.73 ± 27.65	95.45 ± 24.19	108.96 ± 29.70	0.001 *
HDL cholesterol	48.53 ± 11.65	52.07 ± 11.69	44.47 ± 10.25	<0.001 *
Triglycerides	104.80 ± 58.12	92.40 ± 43.63	119.41 ± 69.04	0.003 *
Atherogenic risk index (ARI)	3.74 ± 1.27	3.33 ± 0.90	4.21 ± 1.46	<0.001 *
Body mass index (BMI)	24.38 ± 3.96	23.99 ± 3.73	24.82 ± 4.19	0.198
Waist to hip ratio (WHR)	0.80 ± 0.07	0.77 ± 0.05	0.84 ± 0.07	7.35 × 10^−13^ *

* *p* Value obtained with Student’s *t*-test and Mann–Whitney U test.

**Table 3:** Descriptive categories of serum lipids and anthropometric variables in each sex.

Variable n (%)	Women (n = 92)	Men (n = 80)	*p* Value
**Total cholesterol** Normal (≤200 mg/dL) High (>200 mg/dL)	78 (84.8) 14 (15.2)	58 (72.5) 22 (27.5)	0.060
**LDL cholesterol** Normal (≤100 mg/dL) High (>100 mg/dL)	56 (60.9) 36 (39.1)	34 (42.5) 46 (57.5)	0.02 *
**HDL cholesterol** Normal (Men: >55 mg/dL, Women: >65 mg/dL) Low (Men ≤ 55 mg/dL, Women ≤ 65 mg/dL)	13 (14.1) 79 (85.9)	9 (11.2) 71 (88.8)	0.650
**Triglycerides** High (>150 mg/dL) Normal (≤150 mg/dL)	11 (12.0) 81 (88.0)	23 (28.7) 57 (71.3)	0.007 **
**Atherogenic risk index (ARI)** High (>5) Normal (≤5)	3 (3.3) 89 (96.7)	19 (23.8) 61 (76.2)	<0.001 **
**Body mass index (BMI)** <25 kg/m^2^ ≥25 kg/m^2^	58 (63.0) 34 (37.0)	45 (56.2) 35 (43.8)	0.435
**Waist to hip ratio (WHR)** Normal: Women < 0.86 and Men < 0.90 High: Women ≥ 0.86 and Men ≥ 0.90	87 (94.6) 5 (5.4)	64 (80.0) 16 (20.0)	0.004 **

*p* Value obtained with Fisher’s exact test. * *p* value < 0.05, ** *p* value < 0.01.

**Table 4:** Significant bivariate correlations between personal and psychological variables with ARI in each sex.

Variable	Women (n = 92)	Men (n = 80)
Age	0.479 **	0.518 **
Schooling	0.418 **	0.323 **
With romantic partner	0.107	0.287 **
With children	0.327 **	0.397 **
With job	0.226 *	0.085
Body mass index (BMI)	0.614 **	0.430 **
Waist/hip ratio (WHR)	0.466 **	0.664 **
Frequency of food consumption in excess	0.332 **	−0.067
Autonomy	−0.006	0.242 *

*p* Values obtained with Spearman’s correlation test. * *p* value < 0.05, ** *p* value < 0.01.

**Table 5:** Multivariate regression analysis for ARI in the global sample.

Variable	B	Beta Coefficient	Significance	Tolerance	Change in R^2^
Constant	−5.134	-	0.000	-	-
Waist/hip ratio (WHR)	8.401	0.460	0.000	0.679	0.375
Body mass index (BMI)	0.070	0.219	0.002	0.706	0.030
Autonomy	0.203	0.162	0.008	0.925	0.019
Quality of food intake	−0.057	−0.114	0.056	0.963	0.013

R of the model: 0.661.

**Table 6:** Multivariate regression analysis for ARI in women.

Variable	B	Beta Coefficient	Significance	Tolerance	Change in R^2^
Constant	0.488	-	0.460	-	-
Body mass index (BMI)	0.084	0.345	0.000	0.762	0.303
Age	0.033	0.381	0.000	0.750	0.079
Daily free hours	0.110	0.269	0.001	0.874	0.032
Positive relations with others	−0.207	−0.238	0.005	0.847	0.042
Schooling	0.201	0.163	0.054	0.811	0.020
Assertiveness	−0.164	−0.176	0.041	0.793	0.018
Positive emotions	0.254	0.182	0.038	0.754	0.017
Quality of food intake	−0.049	−0.144	0.075	0.889	0.018

R of the model: 0.728.

**Table 7:** Multivariate regression analysis for ARI in men.

Variable	B	Beta Coefficient	Significance	Tolerance	Change in R^2^
Constant	−8.227	-	0.000	-	-
Waist/hip ratio (WHR)	13.219	0.632	0.000	0.939	0.396
Autonomy	0.441	0.296	0.003	0.726	0.036
Purpose in life	−0.294	−0.228	0.028	0.647	0.021
Emotional regulation	−0.251	0.217	0.018	0.829	0.030
Monthly extra money	−0.168	−0.153	0.085	0.875	0.020

R of the model: 0.710.

## Data Availability

Not applicable.

## Supplementary Materials

The following supporting information can be downloaded at: https://www.mdpi.com/article/10.3390/jpm13101452/s1, Supplementary file S1: Items included in the emotional intelligence subscales of the TEIQUE scale.
